# *Apolipoprotein E* Gene Variants on the Risk of End Stage Renal Disease

**DOI:** 10.1371/journal.pone.0083367

**Published:** 2013-12-13

**Authors:** Cheng Xue, Wei Nie, Dan Tang, Lujiang Yi, Changlin Mei

**Affiliations:** 1 Department of Nephrology, Shanghai Changzheng Hospital, Second Military Medical University, Shanghai, China; 2 Department of Respiratory Disease, Shanghai Changzheng Hospital, Second Military Medical University, Shanghai, China; 3 Department of Gastroenterology, Shanghai Changzheng Hospital, Second Military Medical University, Shanghai, China; 4 Department of Clinical Laboratory, the First Affiliated Hospital of Nanjing Medical University, Nanjing, Jiangsu Province, China; University of Sao Paulo Medical School, Brazil

## Abstract

**Objective:**

End-stage renal disease (ESRD) is a severe health concern over the world. Associations between apolipoprotein E (apoE) gene polymorphisms and the risk of ESRD remained inconclusive. This study aimed to investigate the association between *apoE* gene polymorphisms and ESRD susceptibility.

**Methods:**

Databases including PubMed, Embase, Web of Science and the Cochrane Library were searched to find relevant studies. Meta-analysis method was used synthesize the eligible studies.

**Results:**

Sixteen pertinent case-control studies which included 3510 cases and 13924 controls were analyzed. A signiﬁcant association was found between ε2 allele and the ESRD risk (odds ratio (OR) = 1.30, 95% conﬁdence interval (CI) 1.15–1.46, *P* < 0.0001; *I* ^2^ = 18%, *P* for heterogeneity = 0.24). The ε2ε3, ε2ε4, ε3ε3, ε3ε4, ε4ε4, ε3 and ε4 were not associated with the susceptibility of ESRD. In the subgroup analysis by ethnicity, there was a statistically significant association between ε2ε3 or ε2 allele and ESRD risk in East Asians (OR = 1.66, 95% CI 1.31–2.10, *P* < 0.0001; OR = 1.62, 95% CI 1.31–2.01, *P* < 0.0001, respectively), but not in Caucasians. E2 carriers had higher plasma apoE (mean difference = 16.24 mg/L, 95% CI 7.76-24.73, *P* = 0.0002) than the (ε3 + ε4) carriers in patients with ESRD. The publication bias was not significant.

**Conclusion:**

The ε2 allele of *apoE* gene might increase the risk of ESRD. E2 carriers expressed higher level of plasma apoE in patients with ESRD. More well-designed studies are needed to conﬁrm these associations in the future.

## Introduction

End-stage renal disease (ESRD) is a significant public health problem in the world. The etiology of ESRD is not clear yet. In addition to dyslipidemia, chronic glomerulonephritis, hypertension and diabetes, ESRD is a multifaceted disorder with inherited components playing an important role [1].

Apolipoprotein E (apoE) is a component of lipoprotein, and is one of the key regulatory proteins in cholesterol and lipoprotein metabolism. It is also the structural protein of chylomicron (CM) remnants, low-density lipoprotein (LDL), very low-density lipoprotein (VLDL), and high-density lipoprotein (HDL) [2]. ApoE acts as the ligand of the LDL receptor and apoE receptor, and is synthesized in kidney, liver and adrenal cortex [3]. The *apoE* knockout mice represented hyperlipidemia, accelerated atherogenesis, and glomerulosclerosis-like lesion [3]. ApoE gene polymorphisms and proteins played important roles in the pathogenesis of ESRD [4]. First, patients with high level of apoE2 were more likely to develop into chronic kidney disease (CKD) or even ESRD, because the clearances of VLDL and CM remnants were delayed by apoE2 [5]. Second, ApoE had a high affinity with extracellular glycosaminoglycans which were binded to many growth factors (e.g. tumor growth factor β and platelet-derived growth factor) [6]. ApoE may increase the risk of kidney injury or ESRD by up-regulating the growth factors. Third, lipoprotein glomerulopathy (LPG) was found to be associated with apoEs and the ε2 phenotype [7]. Patients with LPG had a high level of apoE in the serum and capillary thrombosis.

*ApoE* gene locates at 19q13.2, which includes 4 exons and 3 introns. Gene polymorphism was determined by the different amino acid residues at 112 and 158 sites in exon 4. *ApoE* gene polymorphisms include three codominant alleles: ε2, ε3, ε4, which encode 3 protein isoforms (E2, E3 and E4) [8]. ApoE2 had the worst binding affinity with the apoE receptor among them [9]. Among the 3 alleles, either 2 of the alleles can produce 6 different phenotypes in total: 3 homozygotes (ε2ε2, ε3ε3, ε4ε4) and 3 heterozygotes (ε2ε3, ε2ε4, ε3ε4). Allele frequency from high to low is: ε3, ε2, ε4, and ε3ε3 is the most common phenotype in human [10]. It is commonly believed the ε2 allele was associated with the progressive decline of renal function, and ε4 reduced the risk of ESRD [11,12]. In Hubacek et al.’s study [13], patients in hemodialysis had higher frequency of ε2 than the control group (15.9% vs. 12.2%). However, Roussos et al. [14] found patients with ESRD had no difference of ε2, ε3 and ε4 distribution compared with the control group. Feussner et al. also failed to find the association between ε2 and ESRD [15].

The results of the studies remained inconsistent. This meta-analysis was performed to investigate the precise role of *apoE* gene variants on the risk of ESRD.

## Methods

### Information sources and Search

Pubmed, EMBASE, Web of Science and the Cochrane library were all searched (published up to May, 2013). The terms in electronic search included “apoE”, “apolipoprotein E”, “ESRD or end-stage renal disease”, “chronic renal failure”, “dialysis”, “polymorphism or mutation or variant”. In addition, Google Scholar was used to check the references of eligible trials to make sure all studies were included.

### Inclusion and Exclusion Criteria

Studies fulfilling the following selection criteria were included in this meta-analysis: (1) the outcome had to be ESRD; (2) using case-control design, and control group were unrelated people chosen randomly from the same geographic region; (3) genotype distributions should be available for estimating an odds ratio (OR) and 95% conﬁdence interval (CI) in both cases and controls. If one of the following existed, studies were excluded: (1) not relevant to *apoE* polymorphisms or ESRD; (2) study design based on sibling or family pairs; (3) neither genotype frequencies nor number reported; (4) reviews or case reports.

### Study Selection and data extraction

Included studies were independently reviewed by two investigators. Relevant data was extracted into predesigned data collection forms. If there was any discrepancy, two investigators resolved it by discussion or a third author would assess the relevant studies. We collected the following data from each included trial: ﬁrst author’s name, year of publication, country, ethnicity, sample size, age, genotyping method, ESRD treatment, and genotype number in cases and controls.

### Statistical Analysis

The strength of the association between *apoE* polymorphisms and ESRD risk was measured by OR and 95% CI. When *P* value < 0.05, OR was considered statistically signiﬁcant. Continuous outcome data from included trials were analyzed using the mean difference (WMD) and 95% CI. The heterogeneity of identified studies was assessed by *I* ^*2*^ and the Chi square-test based Cochrane Q-test. When *P* value > 0.10 for the Q test, it indicated a lack of heterogeneity among the studies. Then the ﬁxed-effects model was used to pool OR. Otherwise, OR was pooled in the random-effects model. The sources of the heterogeneity and ethnic-specific effect were evaluated by subgroup analyses performed by ethnicity. One-way sensitivity analysis was carried out to access the stability of the meta-analysis. We also excluded the studies not in Hardy-Weinberg equilibrium (HWE) to perform the sensitivity analysis. Cumulative meta-analysis was made by sequential random-effects pooling (starting with the earliest studies), in order to show the consequence of adding studies on the effect size. HWE in controls was tested by the Chi-square test [16]. The Begg’s test and Egger’s test were used to assess publication bias [17]. When performing a series of comparisons in the same sample, the bonferroni correction of critical *P* values was used. Revman 5.1 software (Nordic Cochrane Center, Copenhagen, Denmark) and STATA 11.0 software (Stata Corporation, College Station, TX) were used to perform all the statistical tests.

## Results

**Figure 1:**
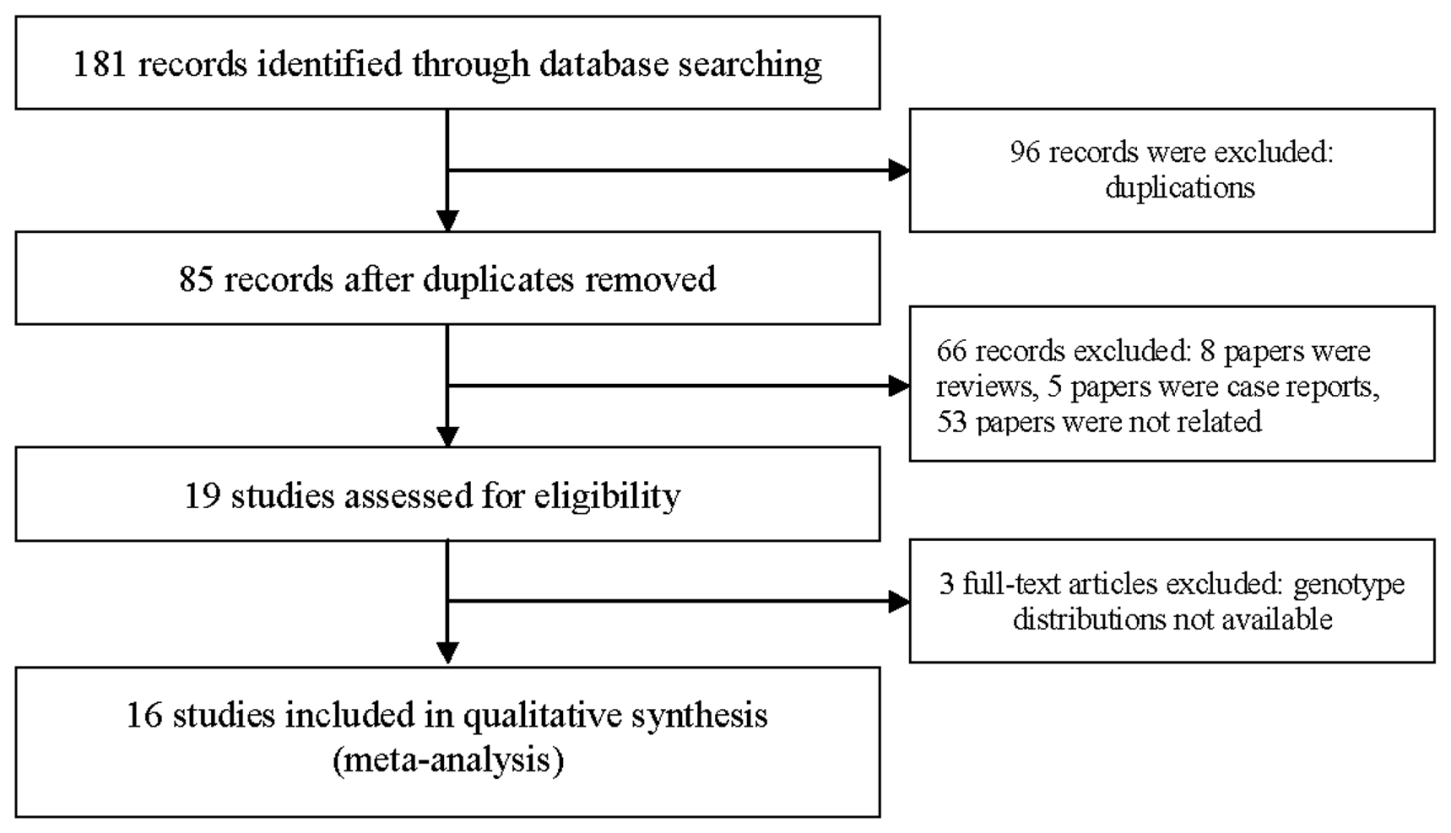
Flow chart of the study identification, inclusion, and exclusion.

A total of 16 clinical studies [14,15,18-31] on ESRD and *apoE* variants published from 1992 and 2008 were identiﬁed, among which seven studies were form Asia [19,22-24,27,29,31], eight from Europe [14,15,20,21,25,26,28,30], and one from North America [18]. The literature review process was shown in Figure **1**. Among the 16 studies, six studies were performed in East Asians [19,22-24,27,29], and 10 in Caucasians [14,15,18,20,21,25,26,28,30,31] (Table **1**). Table **S1** showed the PRISMA 2009 Checklist. Figure **S1** showed the PRISMA 2009 Flow Diagram. Renal replacement therapies in patients with ESRD included continuous ambulatory peritoneal dialysis (CAPD), hemodialysis (HD) or HD plus CAPD. Two included studies used CAPD in the ESRD group [18,22], four using HD plus CAPD [14,24,29,30], and 10 using HD [15,19-21,23,25-28,31]. All the studies were performed in adults. HWE examination results and genotype frequencies were listed in Table **2**. All the studies fit the HWE except three articles [18,23,25].

**Table 1:** Characteristics of included trials.

Author /Year	Country	Ethnicity	Age group	Case (n)	Control (n)	Male/Female of ESRD (n)	Dialysis type	Genotyping method
Feussner/1992	Germany	Caucasian	Adults	560	1031	NA	HD	PCR
Eggertsen/1997	USA	Caucasian	Adults	51	407	25/26	CAPD	PCR
Olmer/1997	France	Caucasian	Adults	66	338	33/33	HD	PCR
Fumitake/1997	Japan	East Asians	Adults	97	173	58/39	HD	PCR
Kohlmeier/1998	Germany	Caucasian	Adults	219	1031	NA	HD	PCR
Choi/1999	Korea	East Asians	Adults	54	194	27/27	CAPD	PCR
Oda/1999	Japan	East Asians	Adults	485	576	NA	CAPD+HD	PCR- RFLP
Imura/1999	Japan	East Asians	Adults	493	422	287/206	HD	PCR
Guz/2000	Turkey	Caucasian	Adults	269	8366	154/115	HD	PCR
Jana/2002	Czech republic	Caucasian	Adults	87	67	53/34	HD	PCR- RFLP
Asakimori/2003	Japan	East Asians	Adults	163	576	85/78	HD	PCR
Roussos/2004	Sweden	Caucasian	Adults	385	407	220/165	CAPD+HD	PCR
Liberopoulos/2004	Greece	Caucasian	Adults	301	200	168/133	HD	PCR
Huang/2005	China	East Asians	Adults	94	108	54/40	CAPD+HD	PCR
Arikan/2007	Turkey	Caucasian	Adults	144	42	49/70	CAPD+HD	PCR
Fahad/2008	Saudi Arabia	Caucasian	Adults	42	50	NA	HD	PCR

CAPD: continuous ambulatory peritoneal dialysis; HD: hemodialysis; PCR, polymerase chain reaction; RFLP, restriction fragment length polymorphism; NA, not available.

**Table 2:** Distribution of *apoE* polymorphism among patients with ESRD and controls.

Studies	ESRD							Control						Hardy–Weinberg equilibrium
	ε2ε2	ε2ε3	ε2ε4	ε3ε3	ε3ε4	ε4ε4		ε2ε2	ε2ε3	ε2ε4	ε3ε3	ε3ε4	ε4ε4	
Feussner/1992	8	60	18	346	117	11		10	124	15	617	236	29	Yes
Olmer/1997	1	8	1	41	15	0		3	40	4	231	54	6	Yes
Fumitake/1997	0	10	2	66	19	1		0	15	0	131	24	3	Yes
Eggertsen/1997	0	2	3	30	14	2		4	42	14	239	65	43	No
Kohlmeier/1998	7	31	1	139	39	2		10	124	15	617	236	29	Yes
Oda/1999	1	50	4	347	82	1		2	35	4	414	111	10	Yes
Choi/1999	0	8	0	37	9	0		0	16	0	147	31	0	Yes
Imura/1999	0	53	0	350	90	0		0	41	0	316	65	0	No
Guz/2000	4	33	2	200	28	2		33	887	67	6208	1079	92	No
Jana/2002	1	9	3	63	11	0		0	9	1	44	12	1	Yes
Asakimori/2003	1	21	1	109	31	0		2	35	4	414	111	10	Yes
Roussos/2004	1	49	9	215	96	15		1	42	9	181	101	9	Yes
Liberopoulos /2004	5	28	3	224	40	1		0	21	2	128	44	5	Yes
Huang/2005	4	30	7	47	6	0		2	15	4	76	11	0	Yes
Arikan/2007	1	18	4	107	12	2		1	3	0	36	2	0	Yes
Fahad/2008	0	4	1	33	3	1		0	3	0	35	11	1	Yes

**Figure 2:**
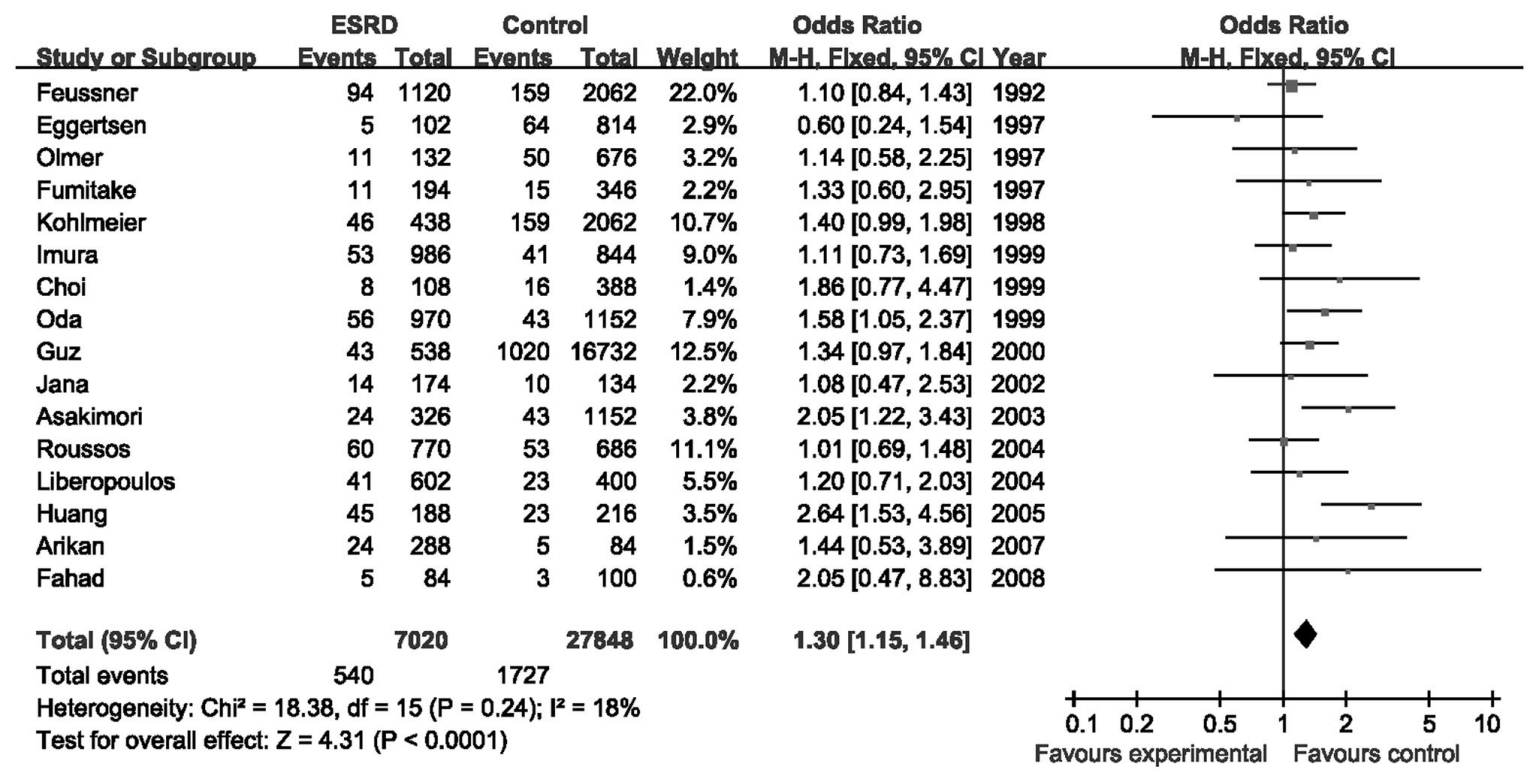
Meta-analysis for the association between the ε2 allele and ESRD risk.

**Figure 3:**
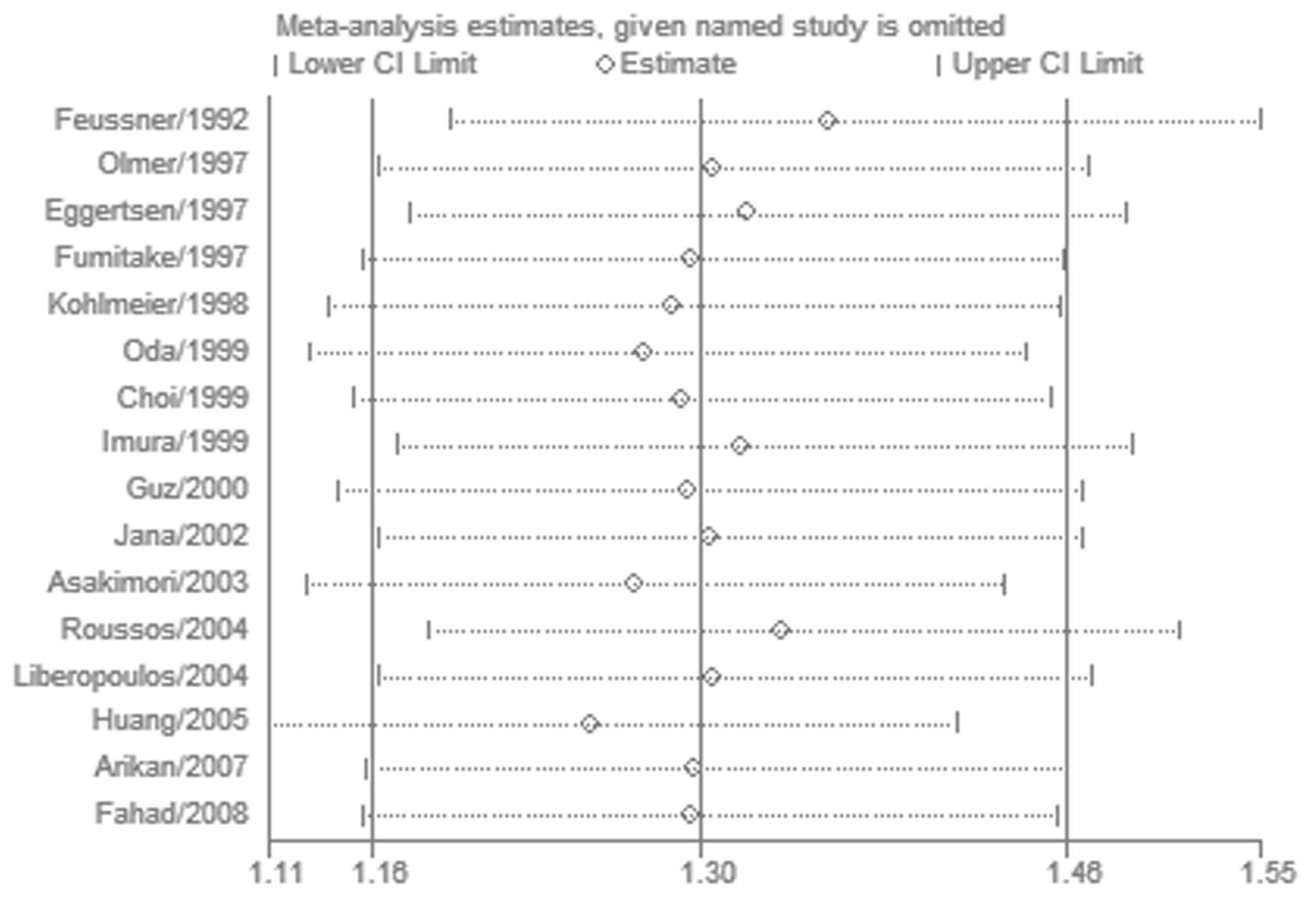
One-way sensitivity analysis for the ε2 allele and ESRD risk.

**Figure 4:**
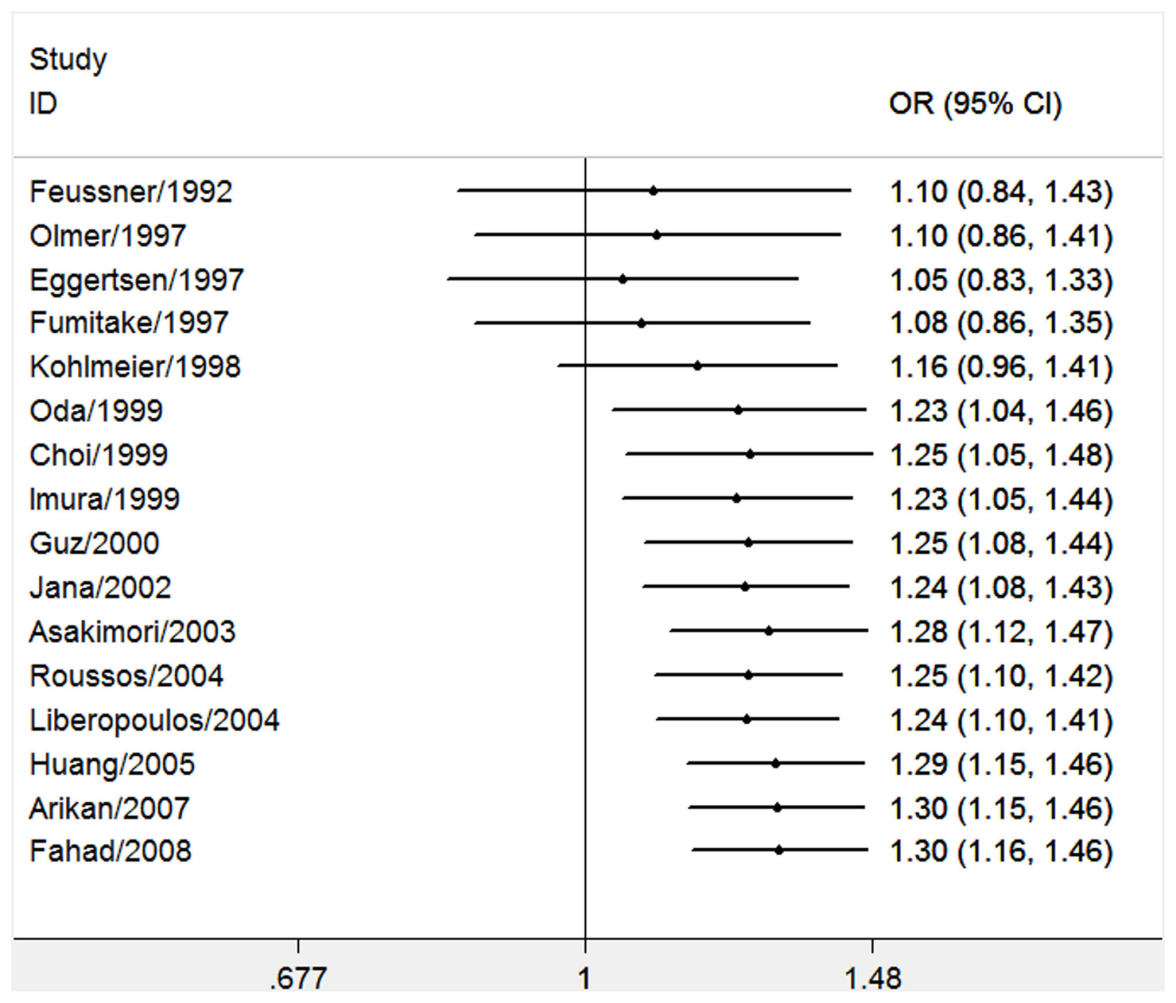
Cumulative meta-analysis of associations between the ε2 allele and ESRD risk.

The eligible studies [14,15,18-31] included 3510 ESRD cases and 13924 controls. According to the bonferroni correction of critical *P* values, the results about *apoE* gene polymorphism were considered to be statistically significant when *P* < 0.00018. A signiﬁcant association was found between ε2 allele and the ESRD risk (OR = 1.30, 95% CI 1.15–1.46, *P* < 0.0001; *I* ^2^ = 18%, *P* for heterogeneity = 0.24) (Figure **2**). The ε2ε3, ε2ε4, ε3ε3, ε3ε4, ε4ε4, ε3 and ε4 were not associated with the susceptibility of ESRD (Table **3**). Interestingly, both ε4ε4 and ε4 allele showed lower risk of ESRD than the control group (OR = 0.55, 95% CI 0.38–0.81, *P* = 0.002; OR = 0.86, 95% CI 0.75–0.99, *P* = 0.04, respectively), but the P values did not reach the statistical criterion. Then in the results of subgroup analysis by ethnicity, there was a statistically significant association between ε2ε3 or ε2 allele and ESRD risk in East Asians [19,22-24,27,29] (OR = 1.66, 95% CI 1.31–2.10, *P* < 0.0001, *P* for heterogeneity = 0.18; OR = 1.62, 95% CI 1.31–2.01, *P* < 0.0001, *P* for heterogeneity = 0.20, respectively) (Table **3**). However, we did not find significant association between ε2 and ESRD in Caucasians (OR = 1.17, 95% CI 1.02–1.36, *P* = 0.03; *I* ^2^ = 0%, *P* for heterogeneity = 0.81) [14,15,18,20,21,25,26,28,30,31]. In consideration of our conserved *P* value, the positive association still could not be excluded in Caucasians. More investigations about Caucasians should be performed in the future. The heterogeneity decreased a lot in the subgroup analysis 1-way sensitivity analysis was performed to evaluate the stability of the meta-analysis of ε2 allele (Figure **3**). When any single study was omitted, the significance of the results did not change. We also conducted the cumulative meta-analyses of ε2 allele. Figure **4** showed the inclination of ε2 allele toward significant association with ESRD risk. Then we excluded the studies [18,23,25] not in HWE in the sensitivity analysis. All the results kept consistent with the primary ones (Table **4**). The heterogeneity of the ε3ε4 was improved (*I* ^2^ = 23%, *P* = 0.21).

**Table 3:** Determination of the genetic effects of *apoE* polymorphisms on ESRD and subgroup analyses.

	Studies number	Heterogeneity (*P* value)	Model	OR (95 % CI)	*P* value
Genetic contrasts					
ε2ε2+ versus ε2ε2-	16	0.78	Fixed	2.03 (1.27, 3.26)	0.003
ε2ε3+ versus ε2ε3-	16	0.06	Random	1.25 (1.03, 1.51)	0.02
ε2ε4+ versus ε2ε4-	16	0.88	Fixed	1.43 (0.99, 2.06)	0.05
ε3ε3+ versus ε3ε3-	16	0.02	Random	0.96 (0.83, 1.10)	0.56
ε3ε4+ versus ε3ε4-	16	0.05	Random	0.91 (0.78, 1.07)	0.27
ε4ε4+ versus ε4ε4-	16	0.41	Fixed	0.55 (0.38, 0.81)	0.002
ε2+ versus ε2 -	16	0.24	Fixed	1.30 (1.15, 1.46)	0.0001
ε3+ versus ε3-	16	0.05	Random	0.97 (0.87, 1.08)	0.57
ε4+ versus ε4-	16	0.06	Random	0.86 (0.75, 0.99)	0.04
East Asians					
ε2ε2+ versus ε2ε2-	6	0.65	Fixed	1.52 (0.47, 4.90)	0.48
ε2ε3+ versus ε2ε3-	6	0.18	Fixed	1.66 (1.31, 2.10)	0.0001
ε2ε4+ versus ε2ε4-	6	0.76	Fixed	1.63 (0.73, 3.65)	0.23
ε3ε3+ versus ε3ε3-	6	0.18	Fixed	0.81 (0.69, 0.94)	0.007
ε3ε4+ versus ε3ε4-	6	0.45	Fixed	1.02 (0.84, 1.23)	0.86
ε4ε4+ versus ε4ε4-	6	0.56	Fixed	0.19 (0.05, 0.73)	0.01
ε2+ versus ε2 -	6	0.20	Fixed	1.62 (1.31, 2.01)	0.0001
ε3+ versus ε3-	6	0.09	Random	0.81 (0.66, 1.00)	0.05
ε4+ versus ε4-	6	0.26	Fixed	0.94 (0.79, 1.11)	0.45
Caucasians					
ε2ε2+ versus ε2ε2-	10	0.64	Fixed	2.16 (1.29, 3.61)	0.003
ε2ε3+ versus ε2ε3-	10	0.75	Fixed	1.01 (0.85, 1.20)	0.90
ε2ε4+ versus ε2ε4-	10	0.72	Fixed	1.38 (0.92, 2.08)	0.12
ε3ε3+ versus ε3ε3-	10	0.33	Fixed	1.11 (0.99, 1.24)	0.09
ε3ε4+ versus ε3ε4-	10	0.04	Random	0.85 (0.68, 1.07)	0.17
ε4ε4+ versus ε4ε4-	10	0.47	Fixed	0.64 (0.43, 0.96)	0.03
ε2+ versus ε2 -	10	0.81	Fixed	1.17 (1.02, 1.36)	0.03
ε3+ versus ε3-	10	0.43	Fixed	1.06 (0.96, 1.17)	0.25
ε4+ versus ε4-	10	0.06	Random	0.81 (0.67, 0.98)	0.03

**Table 4:** Sensitivity analysis of the association between *apoE* polymorphisms and ESRD.

Sensitivity analysis	Studies number	Heterogeneity (*P* value)	Model	OR (95 % CI)	*P* value
ε2ε2+ versus ε2ε2-	13	0.79	Fixed	1.91 (1.13, 3.22)	0.02
ε2ε3+ versus ε2ε3-	13	0.04	Random	1.32 (1.04, 1.66)	0.02
ε2ε4+ versus ε2ε4-	13	0.81	Fixed	1.46 (0.98, 2.17)	0.06
ε3ε3+ versus ε3ε3-	13	0.009	Random	0.96 (0.80, 1.14)	0.64
ε3ε4+ versus ε3ε4-	13	0.21	Fixed	0.85 (0.74, 0.96)	0.01
ε4ε4+ versus ε4ε4-	13	0.30	Fixed	0.57 (0.38, 0.86)	0.007
ε2+ versus ε2 -	13	0.24	Fixed	1.34 (1.17, 1.54)	0.0001
ε3+ versus ε3-	13	0.03	Random	0.96 (0.84, 1.11)	0.61
ε4+ versus ε4-	13	0.08	Random	0.83 (0.71, 0.97)	0.02

**Figure 5:**
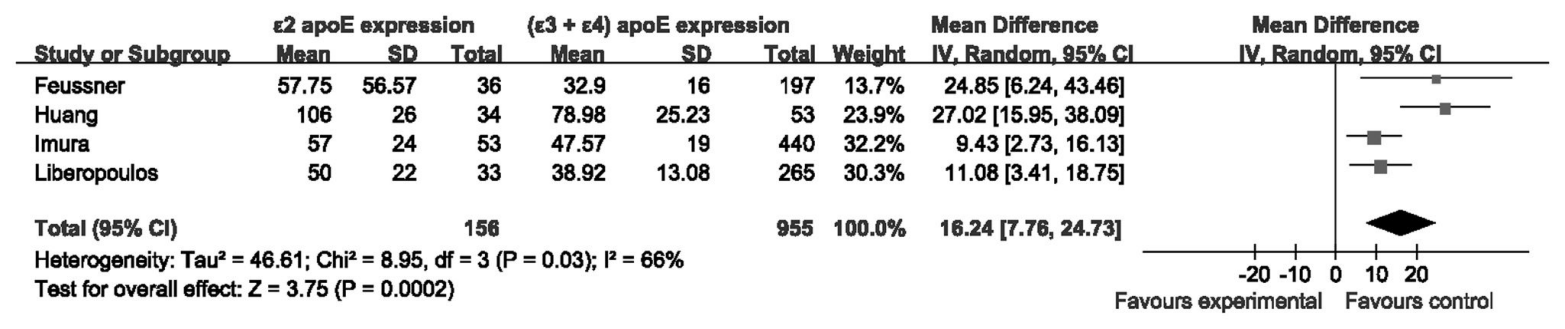
Meta-analysis for the plasma apoE between ε2 carriers and (ε3 + ε4) carriers in the patients with ESRD.

To investigate whether ε2 allele have higher expression of plasma apoE than the (ε3 + ε4) phenotypes in patients with ESRD, we performed another meta-analysis. Ε2 carriers with ESRD had increased apoE expression (4 studies [15,23,28,29], WMD = 16.24 mg/L, 95% CI 7.76-24.73, *P* = 0.0002; *I* ^2^ = 66%; *P* for heterogeneity = 0.03) than the (ε3 + ε4) carriers (Figure **5**).

**Figure 6:**
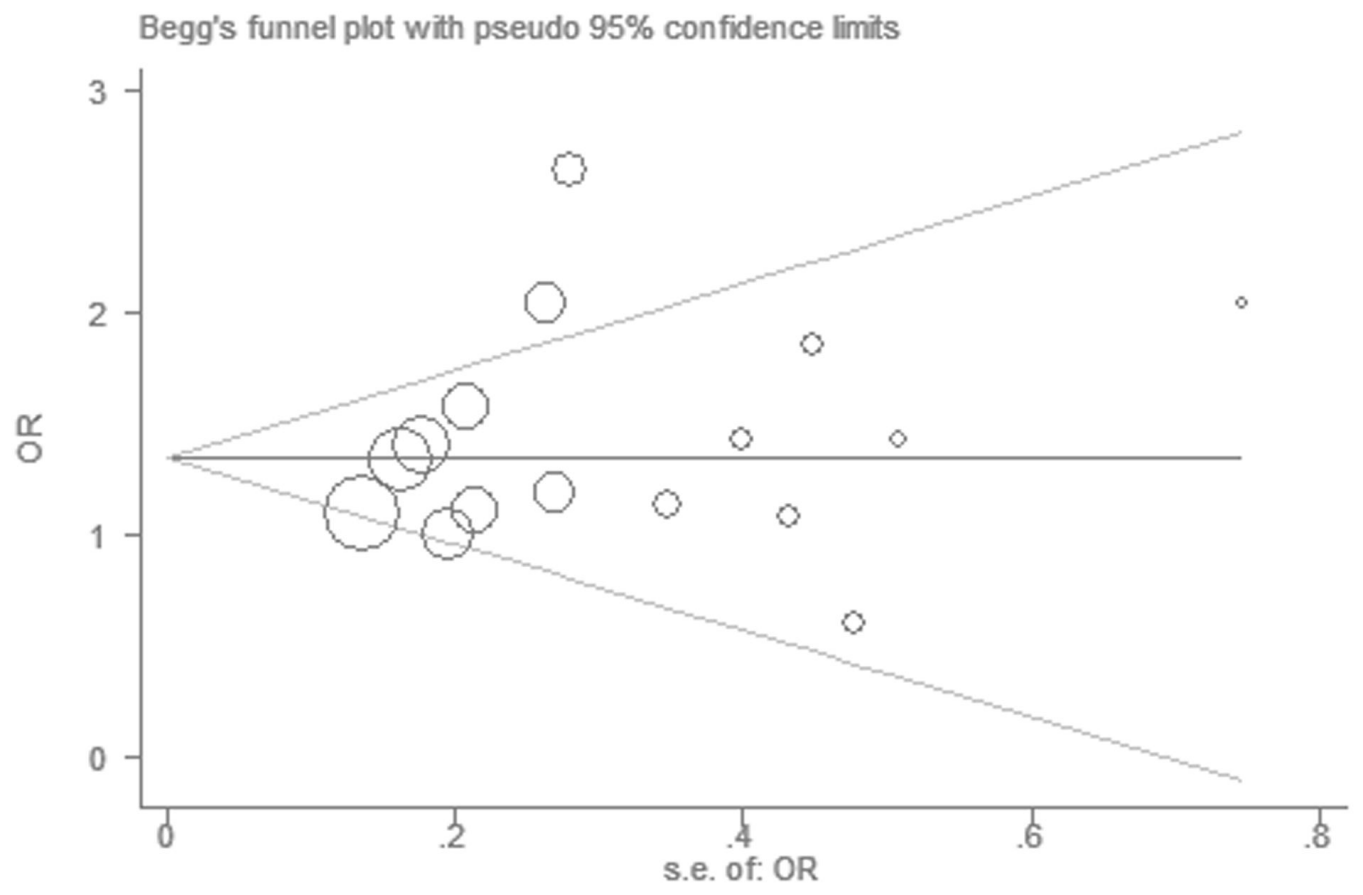
Funnel plot for the ε2 allele and ESRD risk.

There was no significant publication bias in the Begg’s test (*P* = 0.344) and Egger’s test (*P* = 0.352). The funnel plot was symmetrical (Figure **6**).

## Discussion

Several previous studies found the ε2 allele was a possible genetic risk factor for all-cause ESRD. Oda et al. [24] found apoE2 had higher frequency in patients with ESRD, and lower frequency of apoE4 was found. Patients carrying ε2 allele were associated with massive proteinuria [24]. Hubacek et al. found the strength of the OR (ε2) increased with the hemodialysis time of the patients. The ε2 allele may have different functions in different stages of ESRD [13]. Hsu et al. [32] proved *apoE* polymorphism could predict the progression of CKD independently, and increase the risk of early CKD manifestations such as high serum creatinine and macroalbuminuria. Recently, Chu et al. [33] found the ε2 allele was associated with lower levels of continuous GFR in non-Hispanic blacks. By the way, the ε2 allele in patients with type 2 diabetes is a risk factor for development of diabetic nephropathy. In the meta-analysis between *apoE* polymorphism and NS, Zhou et al. [10] found the *apoE* polymorphisms were associated with the susceptibility of NS. The ε2 allele may be a risk factor of renal disease.

In this meta-analysis, the association between the *apoE* polymorphisms and ESRD risk was explored. Sixteen eligible case-control studies which included 3510 cases and 13924 controls were analyzed. The results indicated that individuals with the ε2 allele showed an increased risk of ESRD in the overall population. Compared with those individuals with the ε3 and ε4 alleles, carriers of ε2 genotype had 30% increased risk of ESRD. Previous studies found the ε4 allele tended to be associated with lower odds of ESRD. However, we did not find the association after the meta-analysis. The results of different plasma apoE expression in the patients with ESRD indicated that ε2 carriers had 16.24 mg/L higher expression of plasma apoE than the (ε3 + ε4) carriers. In conclusion, the ε2 allele might increase the risk of ESRD and express more apoE protein. Early screening of the ε2 allele might prevent the patients with CKD from progression into ESRD.

Ε2 allele might affect the susceptibility of ESRD through the lipid and non-lipid mediated mechanisms. First, apoE is mainly secreted by the mesangial cells in kidney [2]. Nonlipid-mediated pathways may be involved in the direct effect on kidney remodeling by apoE [32]. Through induction of matrix heparin sulfate proteoglycan (HSPG), the proliferation of mesangial cell could be differentially inhibited by the apoE’s isoforms [34]. Ε2 shows the least antiproliferative effect on the mesangial cell. More severe histological damage was found among patients with ε2 allele and IgA nephropathy [35]. Additionally, apoE may have isoform-specific effects on vascular smooth muscle proliferation, which may affect progression of ESRD [36]. Second, lipid disorder could accelerate the progression to ESRD. ApoE plays an important role in the modulation of circulating lipid and lipoprotein. The structural basis of apoE protein types are determined by the *apoE* polymorphism [37]. ApoE2 which is produced by the ε2 allele has the lowest binding ability to the receptor. Then the uptaking and clearance of CM or VLDL remnants were impaired in the liver [5]. The ε2 carriers had higher VLDL, TG and apoE2 concentration in the serum, which is associated with type iii hyperlipidemia and kidney disease [38]. The performance the hypertriglyceridemia caused by apoE2 might easily lead to renal vascular atherosclerosis which promote the development of ESRD. 

In the results of subgroup analysis by ethnicity, there was a significant association in East Asians, but not in Caucasians. The findings from races seemed different. The possible reasons included: (1) *apoE* allele frequencies were affected by different genetic backgrounds and geographical diversities; (2) different eating habits may lead to various types of lipid metabolism; (3) the primary kidney disease of ESRD was different, in which the *apoE* allele played different roles. Although the P value of ε2 on ESRD risk in Caucasians was 0.03, a positive association between ε2 and ESRD risk may exist. When studies in Caucasians got larger sample size, we might have sufficient statistical power to detect the slight effect.

 Both cumulative meta-analysis and one-way sensitivity analysis got highly stable results which were in accordance with the primary result of ε2. Publication bias was little which made the results more reliable. In overall populations, moderate heterogeneity was observed for the ε2ε3, ε3ε3, ε3ε4, ε3 and ε4 polymorphisms. Subgroup analysis was used to find the sources of heterogeneity. In the subgroup analysis by ethnicity, the heterogeneity among the comparisons decreased effectively or disappeared. So the main source of heterogeneity might be from different populations. The sensitivity analysis based on studies in HWE [14,15,19-22,24,26-31] found the same results with the ones including all the studies [14,15,18-31]. Different methods for meta-analysis all suggested that ε2 allele might play a role in the etiology of ESRD.

 There were some limitations of this meta-analysis. First of all, the number of available studies included in this meta-analysis was moderate. Only one study provided the apoE gene frequencies in different etiologies of ESRD [14], and the subgroup analysis by etiology was not able to perform, especially about the influences of diabetic nephropathy and non-diabetic nephropathies. More studies not only on all-cause ESRD but also on ESRD with or without diabetic nephropathy could solve this problem in the future. Second, Asians and Caucasians were studied in most of the case-control studies, so the results may be applicable only to these ethnicities. Third, gene-gene and gene-environment interactions could not be addressed in this study because of insufficient data from published studies. Finally, except for the selected databases we have searched, there may be some relevant studies with negative results were missed.

## Conclusion

In conclusion, the ε2 allele of *apoE* gene might increase the risk of ESRD, and expressed more apoE protein. More well-designed studies are needed to prove these associations in the future.

## Supporting Information

Figure S1 **PRISMA 2009 Flow Diagram.**
(TIF)

Table S1 **PRISMA 2009 Checklist.**
(DOC)
