# Supplementary figures and images for: Apolipoprotein E Gene Variants on the Risk of End Stage Renal Disease
Source: PLoS One. 2013 Dec 13;8(12):e83367. doi: 10.1371/journal.pone.0083367 (PMC3862680; doi:10.1371/journal.pone.0083367)

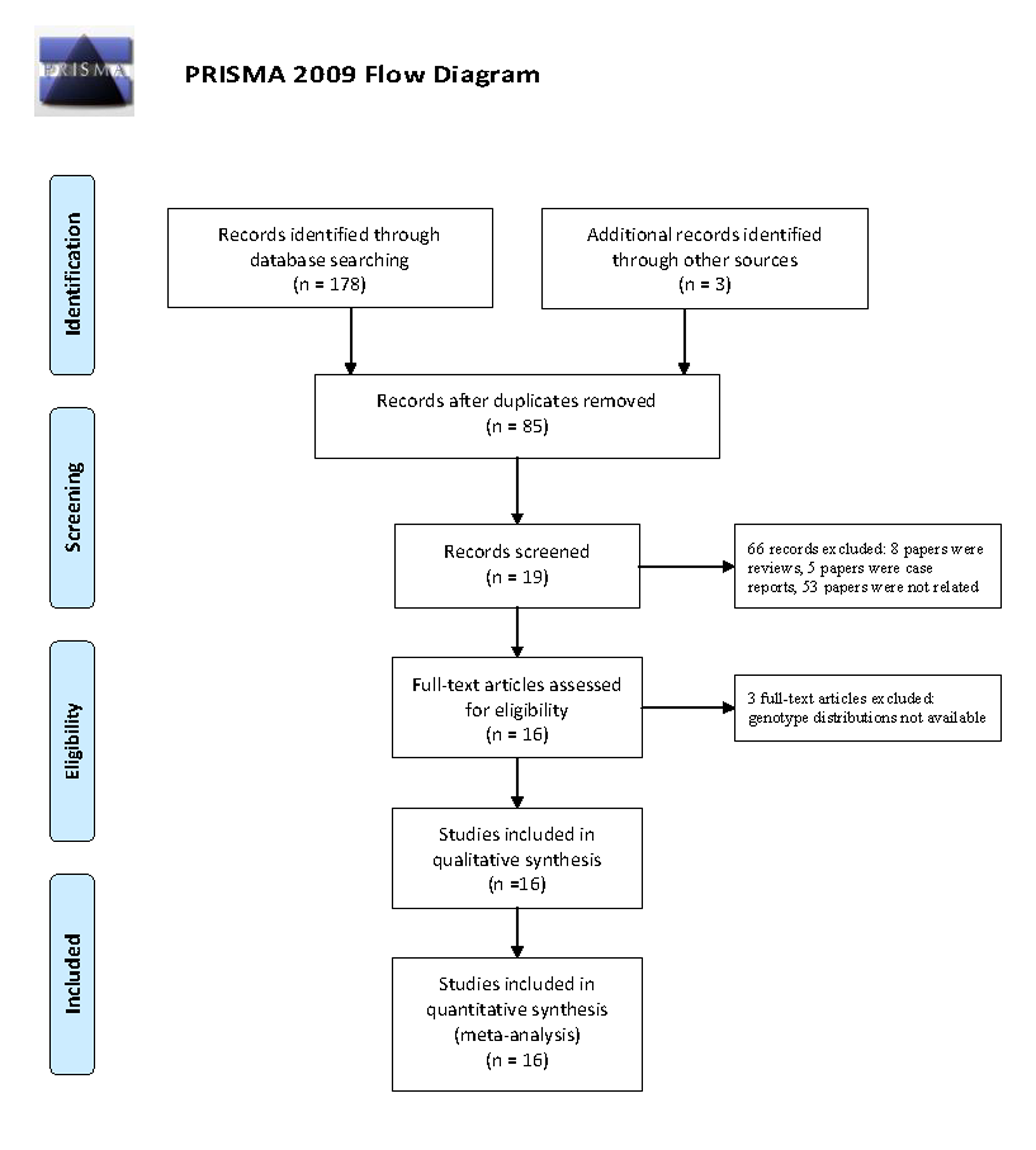

Supplement: Figure S1 — PRISMA 2009 Flow Diagram. (TIF) [file pone.0083367.s001.tif]
